# Prevention and Recovery of COVID-19 Patients With Kampo Medicine: Review of Case Reports and Ongoing Clinical Trials

**DOI:** 10.3389/fphar.2021.656246

**Published:** 2021-06-23

**Authors:** Shin Takayama, Takao Namiki, Hiroshi Odaguchi, Ryutaro Arita, Akito Hisanaga, Kazuo Mitani, Takashi Ito

**Affiliations:** ^1^ Department of Kampo Medicine, Tohoku University Hospital, Sendai, Japan; ^2^ Department of Education and Support for Regional Medicine, Tohoku University Hospital, Sendai, Japan; ^3^ Department of Kampo and Integrative Medicine, Tohoku University Graduate School of Medicine, Sendai, Japan; ^4^ Department of Japanese-Oriental (Kampo) Medicine, Graduate School of Medicine, Chiba University, Chiba, Japan; ^5^ Oriental Medicine Research Center, Kitasato University, Minato-ku, Japan; ^6^ Hospital Bando, Ibaraki, Japan; ^7^ Akashi Clinic Kanda, Chiyoda-ku, Japan; ^8^ Department of Yamato Kampo Medicine and Pharmacy Center, Nara Medical University, Kashihara, Japan; ^9^ Mitani Family Clinic, Osaka, Japan

**Keywords:** COVID-19, SARS-CoV-2, Japan society for oriental medicine, Kampo, treatment, prevention, recovery

## Abstract

Coronavirus disease 2019 (COVID-19) spread to Japan in 2020, where the number of infected patients exceeded 250,000 and COVID-related deaths exceeded 3,500 in one year. Basic guidelines for infection control were implemented in Japan, and research and development of effective drugs and vaccines were promoted. This included considering Kampo medicine, which has a long history of treating recurring emerging viral infections. Considering the characteristics of the disease (inflammation of the upper and lower respiratory tract as well as potential neural damage and vasculitis), Kampo medicine could be considered as a treatment strategy due to its antiviral and anti-inflammatory effects induced by multiple active substances that could aid in disease prevention and recovery. In this study, case reports on the management of COVID-19 with Kampo medicine, which were published until March 31, 2021, were reviewed. The search strategy involved the use of Medline and hand-searching. Twenty two patients were treated using Kampo medicines with or without Western medicine, based on individual conditions. On the other hand, the effects of Kampo medicines as a potential preventive treatment (pre-infection), active treatment (especially in the acute and subacute stage), or treatment of sequelae to aid recovery (after infection) in the different stages of COVID-19 are being studied as research projects in the Japan Society for Oriental Medicine (JSOM). JSOM has also organized a pioneering project of clinical trials for COVID-19, some of which are now in progress.

## Introduction

### Spread of Coronavirus Disease 2019 (COVID-19) in Japan

The mass occurrence of an undefined pneumonia was first reported in Wuhan, China, to the World Health Organization (WHO) on December 31, 2019 (World Health Organization, 2019). Thereafter, the cause of this pneumonia was reported to be severe acute respiratory syndrome coronavirus 2 (SARS-CoV-2) and the disease was named coronavirus disease 2019 (COVID-19). COVID-19 rapidly spread worldwide, including Asia, in a year. At the end of March 2021, the number of confirmed COVID-19 patients were 90,159 in China, 474,641 in Japan, and 103,639 in Korea, with the number of deaths being 4,636 in China, 9,155 in Japan, and 1,735 in Korea; these numbers have been increasing.

**FIGURE 1:**
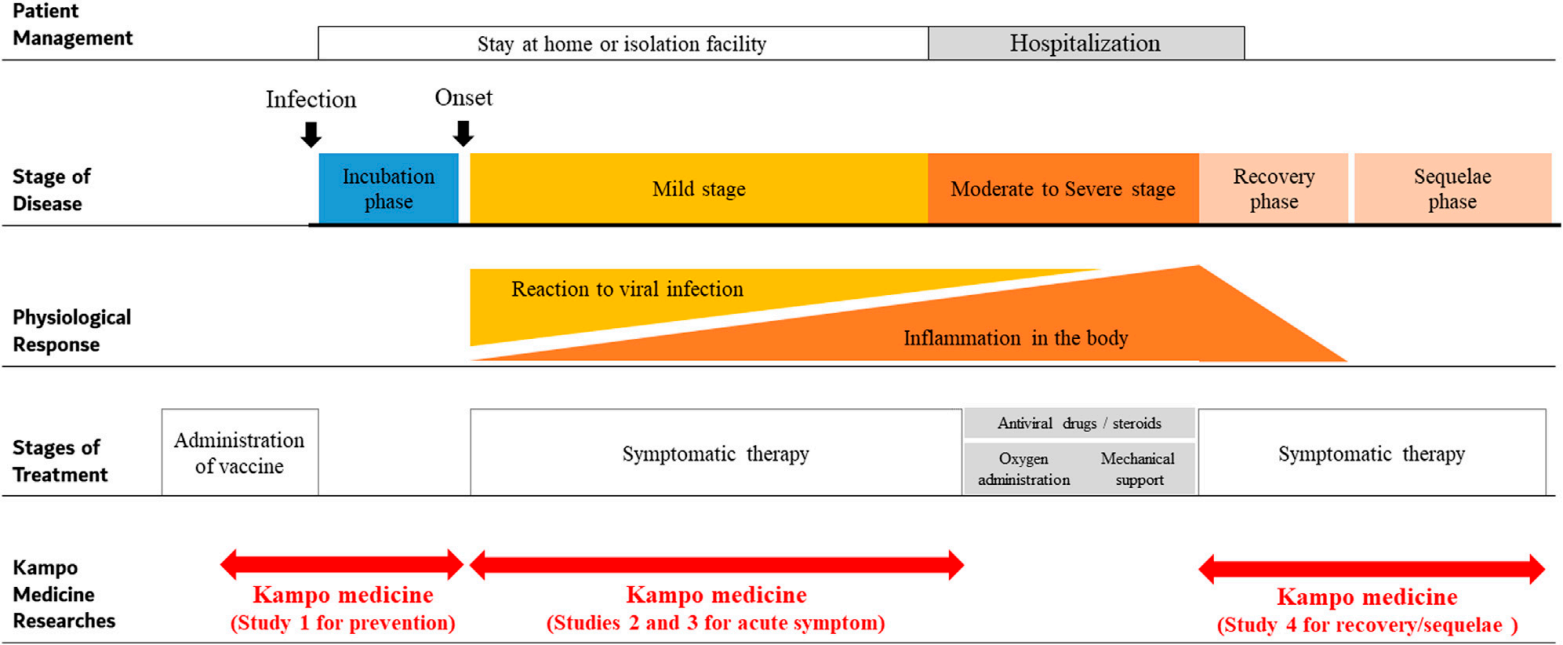
Concept and clinical trials of Kampo medicine for COVID-19.

The first case of COVID-19 in Japan was confirmed on January 15, 2020. The Japanese government was quick to classify COVID-19 as a serious infectious disease on February 1, 2020. Following which, basic guidelines for infection control against COVID-19 were published on February 25, 2020 (Ministry of Health, Labour and Welfare of Japan, 2020a). The number of patients continued to increase, thus increasing the burden on hospitals, and creating a shortage of beds due to many patients requiring hospitalization. Efforts were made to follow patients via course observation at home in asymptomatic cases, while mild stage patients were cared for in isolation facilities (Figure 1). On March 11, 2020, the WHO declared COVID-19 global pandemic based on the spread and severity of the infection (World Health Organization, 2020). Additional guidance for basic infection control against COVID-19 in Japan was published on March 28, 2020, stating to avoid “enclosed and dense spaces and close contact with others.” In addition, the guide stated that ensuring the healthcare system and promoting research and development of effective drugs and vaccines for COVID-19 was important (National Institute of Infectious Diseases, 2020).

### History of an Emerging Viral Infection as a Pandemic

The Spanish flu, a global pandemic, affected Japan from 1918 to 1919, and is now known to be caused by the H1N1 influenza virus. Its fatality rate was reported to be approximately 2.5% worldwide and 1.6% in Japan (Influenza, 2008). In Japan, 23.8 million people had been infected and 388,000 people had died by the end of 1919. Its symptoms are similar to that of a common cold, and include fever, headache, muscle aches, and joint pain; however, it spreads to the lungs and causes severe pneumonia in severe cases. During this period, famous Japanese Kampo doctors treated patients with the Spanish flu using Kampo medicines. Saikatsugekito, daiseiryuto, chikujountanto, kososan, shoseiryuto, shomakakkonto, and additional or reductional formulae were applied according to the patient’s symptoms (Yasui 2007; Irie and Nakae, 2020).

Additionally, SARS-CoV in 2003 (World Health Organization, 2015), influenza A (H1N1) pdm09 in 2009, Middle East respiratory syndrome (MERS)-CoV in 2012 (Zaki et al., 2012), and SARS-CoV-2 in 2019 have affected the world, and the contribution of Kampo medicine for the treatment of these emerging viral infections need to be evaluated using clinical studies. According to a clinical trial, Nabeshima et al. reported the effects of maoto on seasonal influenza compared with neuraminidase inhibitors and found that the time to fever resolution was shorter with maoto than with oseltamivir in a randomized controlled trial (RCT) (Nabeshima et al., 2012). Yoshino et al. reported that maoto may decrease the duration of fever when used alone or in combination with neuraminidase inhibitors in a systematic review (Yoshino et al., 2019). Arita et al. described pharmacological activities of saikatsugekito against viral infection and respiratory inflammation. In this review, some components of saikatsugekito demonstrated therapeutic effect in the infection processes of single-strand RNA viruses. The therapeutic effect encompasses the enhancement of the immunomodulating activities against experimental inflammation, including cytokine production, regulation of immune cells, and protection against lung tissue injury (Arita et al., 2020). This information could be useful for developing a treatment approach to target COVID-19.

### Features of SARS-CoV-2 and COVID-19

Coronavirus is a single-stranded RNA virus. Six coronaviruses, especially SARS and MERS, cause severe pneumonia. The other four types of coronaviruses are known to cause symptoms more akin to the common cold. SARS-CoV-2 is genetically similar to SARS-CoV and causes severe pneumonia and various related symptoms (Lu et al., 2020). SARS-CoV-2 infects humans through the human angiotensin-converting enzyme two receptor, which is located at the surface of the nasal mucosa and type 2 lung epithelial cells (Hoffmann et al., 2020; Zhou et al., 2020).

The infectious period is from two days before onset to 7–10 days after onset, and the infectivity is extremely high just before and shortly after onset (He et al., 2020). The main route of infection is via airborne droplet transmission; however, contact transmission through patients or contaminated surfaces is also contributory. Of note, it has been confirmed that erosols can be generated and cause infection (Jarvis, 2020).

The typical clinical course of COVID-19 is shown in Figure 1. After infection with SARS-CoV-2, an incubation period of 1–14 days (mean 5.8 days) occurs before onset of common cold symptoms (Wu and McGoogan 2020). As per a study, among the sources of infection, 45% of individuals are infectious before the onset of symptoms and 5% are infectious and completely asymptomatic (Ferretti et al., 2020). The source of infection was also reported to be asymptomatic individuals in 50% of the cases, while 40% of the cases were from symptomatic individuals and 10% came from environmental sources, i.e., contact transmission (Ferretti et al., 2020). Another study indicated that approximately 80% of the patients in the mild stage recovered from the disease; however, 20% of the patients required hospitalization, of which 5% required treatment in the intensive care unit (Wu and McGoogan 2020). The following criteria were used to determine staging: mild stage, SpO_2_ ≥ 96% with cough without dyspnea; moderate I stage, SpO_2_ 93–96% with dyspnea and pneumonia findings; moderate II stage, SpO2 ≤ 93% and required oxygen administration and treatment; and severe stage, required intensive care or mechanical ventilation. Age (≥65 years) and underlying diseases such as chronic respiratory disease, chronic renal disease, and diabetes mellitus are risk factors for severe disease in COVID-19 patients. Significantly, in severe-stage COVID-19 patients, levels of interleukin-6, interleukin-1beta, and soluble tumor necrosis factor receptor one are elevated, suggesting that a strong inflammatory response is related to severe disease (McElvaney et al., 2020). Symptoms persist even after stabilization of patients and can be problematic. Post-acute COVID-19 follow-up showed that 87.4% of patients complained of symptoms, especially fatigue (53.1%), dyspnea (43.4%), joint pain (27.3%), and chest pain (21.7%) even 60 days after the onset of COVID-19 in one study (Carfì et al., 2020).

### Vaccine for COVID-19

In Japan, an agreement to obtain a supply of the mRNA vaccine with Pfizer Inc. and the adenovirus vector vaccine with AstraZeneca Inc. was announced on July 31 and August 7, 2020, respectively.

### Treatment of COVID-19

In Japan, remdesivir was approved on May 7, 2020, and dexamethasone was approved on July 21, 2020, for the treatment of COVID-19. Remdesivir and dexamethasone were approved for patients in the moderate to severe stage of COVID-19; however, favipiravir, ciclesonide, or lopinavir-ritonavir were not approved as of December 31, 2020.

### Review of Clinical Reports on Kampo Treatment for COVID-19

Case reports on the management of COVID-19 using Kampo medicine, published until March 31st, 2021, were reviewed. The search strategy involved the use of Medline and hand-searching. In 2020 and 2021, several case reports and case series of patients with COVID-19 treated with Kampo medicines were reported (Table 1). Kampo medicine listed in Table 1 are described in detail in Table 2. Plant names and part of each ingredient composed in Kampo medicine are listed in Supplementary Table S1.

**TABLE 1:** Review of clinical reports on the management of COVID-19 with Kampo medicine.

Author	Cases		Age (y.o.)/gender	Pre-existing conditions	Symptoms	Stage in COVID-19	Treatment			Outcome
	No.	Diagnosis					Oxygen administration	Others	Kampo medicine	
Niitsuma et al. (2020)	1	Pneumonia	78/M	Post stroke, hypertension	Fever, dyspnea, malaise, appetite loss	Moderate II	Yes	Lopinavir/ritonavir, hydrocortisone, ceftriaxone, sulbactam/ampicillin, acetaminophen	maoto, daiseiryuto, chikujountanto	Improved
	2	Pneumonia	74/F	None	Joint pain, fever, appetite loss	Moderate I	No	Baloxavir marboxil, moxifloxacin, lopinavir/ritonavir, warfarin	maoto, keishito + eppikajutsuto	Improved
Kashima et al. (2020)	3	Pneumonia	50/F	None	Fever, malaise, headache, block nose, lumbago, appetite loss	Moderate I	No	Acetaminophen, ciclesonide, favipiravir	kakkonto + shosaikoto, shosaikoto + bukuryoingohangekobokuto	Improved
	4	Pneumonia	53/F	Systemic lupus erythematosus treated with prednisolone	Cold and heat sensation, malaise, cough, lumbago, appetite loss, diarrhea	Moderate II	Yes	Ceftriaxone, azithromycin, acetaminophen, ciclesonide, favipiravir	shosaikoto, saikanto, chikujountanto, bukuryoingohangekobokuto, jinsoin, hochuekkito, gokoto, kikyosekko, keishibukuryogan, saffron; combination of the above according to the condition	Improved
Homma et al. (2020a)	5	Pneumonia	30/M	Atopic dermatitis	Headache, fever, throat pain, coldness, cough, malaise	Moderate II	Yes	Acetaminophen	bakumondoto + hangekobokuto	Improved
Homma et al. (2020b)	6	Pneumonia with facial paralysis and olfactory disturbance	35/F	None but smoker	Cough, malaise, sore throat, nausea, fever, headache, smell impairment, taste impairment, facial paralysis	Moderate II	Yes	Acetaminophen, ciclesonide, favipiravir	maoto	Improved
Irie et al. (2020)	7	Common cold with taste disorder	41/F	N/A	Headache, sore throat, nausea, taste disorder, fever	Mild	No	None	kakkonto + shosaikotokakikyosekko	Improved
	8	Common cold with taste disorder	16/F	N/A	Nasal congestion, taste disorder	Mild	No	None	kakkonto + shosaikotokakikyosekko	Improved
	9	Common cold with taste disorder	12/M	N/A	Nasal congestion, taste disorder	Mild	No	None	kakkonto + shosaikotokakikyosekko	Improved
Watanabe and Watanabe. (2020)	10	Common cold with smell impairment	59/M	None	Fever, sore through, smell impairment, abdominal fullness	N/A	No	Acetaminophen, clarithromycin, tranexamic acid, L-carbocisteine	Qing Fei Pai Du Tang, hochuekkito	Improved
	11	Common cold	36/F	Hashimoto’s disease	Fever, cough, sputum, headache, nasal bleeding	N/A	No	Acetaminophen, clarithromycin, tranexamic acid, L-carbocisteine	Qing Fei Pai Du Tang, kakkoshokisankakikukakyouninrengyohakka, hochuekkito	Improved
Kyo (2020)	12	Pneumonia	40/F	N/A	High fever, cough, dyspnea, chest pain, chest oppression, malaise, headache	Moderate II	Yes	Intravenous hydration	saikokeishito, hangekobokuto, maobushisaishinto	Improved
	13	Pneumonia	64/F	N/A	High fever, cough, dyspnea, chest pain, chest oppression, malaise, headache	Moderate II	Yes	Intravenous hydration	saikokeishito, hangekobokuto, maobushisaishinto	Improved
	14	Pneumonia	47/M	N/A	High fever, dyspnea, chest pain, malaise	Moderate II	Yes	Intravenous hydration	saikatsugekitogokoto, daiseiryuto	Improved
Yamasaki (2020)	15	Pneumonia	67/F	Post operation of lung cancer	Fever, malaise	Moderate I	No	—	makyokansekito + ireito + shosaikotokakikyosekko	Improved
	16	Pneumonia	69/M	Type II diabetes mellitus	Fever, cough	Moderate I	No	Favipiravir	makyokansekito + ireito + shosaikotokakikyosekko	Improved
Takayama et al. (2021b)	17	Pneumonia	52/M	None	Cough, sputum, joint pain	Moderate I	No	None	gokoto	Improved
Takayama et al. (2021a)	18	Common cold with smell and taste impairment	36/F	None	Nasal discharge, blocked nose, smell and taste impairment	N/A	No	None	kakkontokasenkyushin'i	Improved
	19	Common cold with smell and taste impairment	18/F	None	Nasal discharge, smell and taste impairment	N/A	No	None	kakkontokasenkyushin’i	Improved
	20	Common cold with smell and taste impairment	24/F	None	Blocked nose, smell and taste impairment	N/A	No	None	kakkontokasenkyushin’i	Improved
	21	Common cold with smell and taste impairment	44/M	None	Blocked nose, smell and taste impairment	N/A	No	None	kakkontokasenkyushin’i	Improved
	22	Common cold and diarrhea with smell and taste impairment	24/M	None	Nasal discharge, blocked nose, cough, sputum, diarrhea, fatigue, smell and taste impairment	N/A	No	None	kakkontokasenkyushin’i, makyokansekito, saireito	Improved

Notes: M; male, F; female. N/A; not assigned.

Mild stage; SpO_2_ ≧ 96% with cough without dyspnea. Moderate I stage; 93% < SpO_2_ < 96% with dyspnea and pneumonia findings. Moderate II stage; 93% ≦ SpO_2_ needed oxygen administration, treatment with steroid and antiviral agent. Severe stage; needed intensive care or mechanical ventilation.

**TABLE 2:** Kampo medicine and Chinese medicine described in the review of case reports.

Japanese names in roman characters	Chinese characters	Chinese name for Pinyin	Ingredients and daily dosage (JP: The Japanese Pharmacopoeia)
Bakumondoto	麦門冬湯	Mai men dong tang	JP Ophiopogon Tuber 10.0 g, JP Brown Rice 5.0 g, JP Pinellia Tuber 5.0 g, JP Jujube 3.0 g, JP Glycyrrhiza 2.0 g, JP Ginseng 2.0 g
Bukuryoingohangekobokuto	茯苓飲合半夏厚朴湯	Fu ling yin he ban xia hou po tang	JP Pinellia Tuber 6.0 g, JP Poria Sclerotium 5.0 g, JP Atractylodes Lancea Rhizome 4.0 g, JP Magnolia Bark 3.0 g, JP Citrus Unshiu Peel 3.0 g, JP Ginseng 3.0 g, JP Perilla Herb 2.0 g, JP Immature Orange 1.5 g, JP Ginger 1.0 g
Chikujountanto	竹筎温胆湯	Zhu ru wen dan tang	JP Pinellia Tuber 5.0 g, JP Bupleurum Root 3.0 g, JP Ophiopogon Tuber 3.0 g, JP Poria Sclerotium 3.0 g, JP Platycodon Root 2.0 g, JP Immature Orange 2.0 g, JP Cyperus Rhizome 2.0 g, JP Citrus Unshiu Peel 2.0 g, JP Coptis Rhizome 1.0 g, JP Glycyrrhiza 1.0 g, JP Ginger 1.0 g, JP Ginseng 1.0 g, Bamboo Culm 3.0 g
Daiseiryuto	大青竜湯	Da qing long tang	Application with Kampo medicine combination i. e. maoto + keishito, eppikajutsuto + keishito
Eppikajutsuto	越婢加朮湯	Yue bi jia zhu tang	JP Gypsum 8.0 g, JP Ephedra Herb 6.0 g, JP Atractylodes Lancea Rhizome 4.0 g, JP Jujube 3.0 g, JP Glycyrrhiza 2.0 g, JP Ginger 1.0 g
Gokoto	五虎湯	Wu hu tang	JP Gypsum 10.0 g, JP Apricot Kernel 4.0 g, JP Ephedra Herb 4.0 g, JP Mulberry Bark 3.0 g, JP Glycyrrhiza 2.0 g
Hangekobokuto	半夏厚朴湯	Ban xia hou po tang	JP Pinellia Tuber 6.0 g, JP Poria Sclerotium 5.0 g, JP Magnolia Bark 3.0 g, JP Perilla Herb 2.0 g, JP Ginger 1.0 g
Hochuekkito	補中益気湯	Bu zhong yi qi tang	JP Astragalus Root 4.0 g, JP Atractylodes Lancea Rhizome 4.0 g, JP Ginseng 4.0 g, JP Japanese Angelica Root 3.0 g, JP Bupleurum Root 2.0 g, JP Jujube 2.0 g, JP Citrus Unshiu Peel 2.0 g, JP Glycyrrhiza 1.5 g, JP Cimicifuga Rhizome 1.0 g, JP Ginger 0.5 g
Ireito	胃苓湯	Wei ling tang	JP Magnolia Bark 2.5 g, JP Atractylodes Lancea Rhizome 2.5 g, JP Alisma Tuber 2.5 g, JP Polyporus Sclerotium 2.5 g, JP Citrus Unshiu Peel 2.5 g, JP Atractylodes rhizome 2.5 g, JP Poria sclerotium 2.5 g, JP Cinnamon Bark 2.0 g, JP Ginger 1.5 g, JP Jujube 1.5 g, JP Glycyrrhiza 1.0 g
Jinsoin	参蘇飲	Shen su tang	JP Pinellia Tuber 3.0 g, JP Poria Sclerotium 3.0 g, JP Pueraria Root 2.0 g, JP Platycodon Root 2.0 g, JP Peucedanum Root 2.0 g, JP Citrus Unshiu Peel 2.0 g, JP Jujube 1.5 g, JP Ginseng 1.5 g, JP Glycyrrhiza 1.0 g, JP Immature Orange 1.0 g, JP Perilla Herb 1.0 g, JP Ginger 0.5 g
Kakkonto	葛根湯	Ge gen tang	JP Pueraria Root 4.0 g, JP Jujube 3.0 g, JP Ephedra Herb 3.0 g, JP Glycyrrhiza 2.0 g, JP Cinnamon Bark 2.0 g, JP Peony Root 2.0 g, JP Ginger 2.0 g
Kakkontokasenkyushin'i	葛根湯加川芎辛夷	Ge gen tang jia chuan xiong xin yi	JP Pueraria Root 4.0 g, JP Jujube 3.0 g, JP Ephedra Herb 3.0 g, JP Glycyrrhiza 2.0 g, JP Cinnamon Bark 2.0 g, JP Peony Root 2.0 g, JP Magnolia Flower 2.0 g, JP Cnidium Rhizome 2.0 g, JP Ginger 1.0 g
Kakkoshokisankagen	霍香正気散加減	Huo xiang zheng qi san jia jian	JP Atractylodes Rhizome 3.0 g, JP Pinellia Tuber 3.0 g, JP Poria Sclerotium 3.0 g, JP Magnolia Bark 2.0 g, JP Citrus Unshiu Peel 2.0 g, JP Platycodon Root 1.5 g, JP Angelica Dahurica Root 1.5 g, JP Perilla Herb 1.0 g, JP Pogostemon Herb 1.0 g, Areca Pericarp 1.0 g, JP Jujube 1.0 g, JP Glycyrrhiza 1.0 g, JP Ginger 0.5 g, JP Chrysanthemum Flower 3.0 g, JP Apricot Kernel 3.0 g, JP Forsythia Fruit 3.0 g, JP Mentha Herb 2.0 g
Keishibukuryogan	桂枝茯苓丸	Gui zhi fu ling wan	JP Cinnamon Bark 3.0 g, JP Peony Root 3.0 g, JP Peach Kernel 3.0 g, JP Poria Sclerotium 3.0 g, JP Moutan Bark 3.0 g
Keishito	桂枝湯	Gui zhi tang	JP Cinnamon Bark 4.0 g, JP Peony Root 4.0 g, JP Jujube 4.0 g, JP Glycyrrhiza 2.0 g, JP Ginger 1.5 g
Kikyosekko	桔梗石膏	Jie geng shi gao	JP Gypsum 10.0 g, JP Platycodon root 3.0 g
Makyokansekito	麻杏甘石湯	Ma xing gan shi tang	JP Gypsum 10.0 g, JP Apricot Kernel 4.0 g, JP Ephedra Herb 4.0 g, JP Glycyrrhiza 2.0 g
Maobushisaishinto	麻黄附子細辛湯	Ma huang fu zi xi xin tang	JP Ephedra Herb 4.0g, JP Asiasarum Root 3.0g, JP Powdered Processed Aconite Root 1.0 g
Maoto	麻黄湯	Ma huang tang	JP Apricot Kernel 5.0 g, JP Ephedra Herb 5.0 g, JP Cinnamon Bark 4.0 g, JP Glycyrrhiza 1.5 g
Renkaseiun	連花清瘟	Lianhua qingwen	Forsythia Fruit (Lianqiao) 170 g, Lonicera Flower (Jinyinhua) 170 g, Ephedra Herb (Mahuang) 57 g, Bitter Apricot Seed (Kuxingren) 57 g, Gypsum (Shigao) 170 g, Indigo Woad Root (Banlangen) 170 g, Male Fern Rhizome (Mianmaguanzhong) 170 g, Heartleaf Houttuynia Herb (Yuxingcao) 170 g, Pogostemon Herb (Guanghuoxiang) 57 g, Chinese Rhubarb Rhizome (Dahuang) 34 g, Roseroot (Hongjingtian) 57 g, Mentha haplocalyx Herb (Bohe) 5 g, Glycyrrhiza Root (Gancao) 57 g
Saikanto	柴陥湯	Chai xian tang	JP Bupleurum Root 5.0 g, JP Pinellia Tuber 5.0 g, JP Scutellaria Root 3.0 g, JP Jujube 3.0 g, JP Ginseng 2.0 g, JP Coptis Rhizome 1.5 g, JP Glycyrrhiza 1.5 g, JP Ginger 1.0 g, Trichosanthes Seed 3.0 g
Saikatsugekito	柴葛解肌湯	Chai ge jie ji tang	Application with Kampo medicine combination i. e. kakkonto + shosaikotokakikyosekko
Saikokeishito	柴胡桂枝湯	Chai hu gui zhi tang	JP Bupleurum Root 5.0 g, JP Pinellia Tuber 4.0 g, JP Scutellaria Root 2.0 g, JP Glycyrrhiza 2.0 g, JP Cinnamon Bark 2.0 g, JP Peony Root 2.0 g, JP Jujube 2.0 g, JP Ginseng 2.0 g, JP Ginger 1.0 g
Saireito	柴苓湯	Chai ling tang	JP Bupleurum Root 7.0 g, JP Alisma Tuber 5.0 g, JP Pinellia Tuber 5.0 g, JP Scutellaria Root 3.0 g, JP Atractylodes Lancea Rhizome 3.0 g, JP Jujube 3.0 g, JP Polyporus Sclerotium 3.0 g, JP Ginseng 3.0 g, JP Poria Sclerotium 3.0 g, JP Glycyrrhiza 2.0 g, JP Cinnamon Bark 2.0 g, JP Ginger 1.0 g
Seihaihaidokuto	清肺排毒湯	Qing fei pai du tang	Original Chinese formula
			Ephedra Herb (Mahuang) 9.0 g, Glycyrrhiza Root and Rhizome processed with honey (Zhigancao) 6.0 g, Apricot Seed (Xingren) 9.0 g, Gypsum (Shengshigao) 15.0–30.0 g, Cinnamon Twig (Guizhi) 9.0 g, Alisma Tuber (Zexie) 9.0 g, Polyporus Sclerotium (Zhuling) 9.0 g, Atractylodes Rhizome (Baizhu) 9.0 g, Poria Sclerotium (Fuling) 15.0 g, Bupleurum Root (Chaihu) 16.0 g, Scutellaria Root (Huangqin) 6.0 g, Pinellina Rhizome processed with ginger (Jiangbanxia) 9.0 g, Ginger (Shengjiang) 9.0 g, Aster Root (Ziwan) 9.0 g, Common Coltsfoot Flower (Kuandonghua) 9.0 g, Blackberrylily Rhizome (Shegan) 9.0 g, Asiasarum Root and Rhizome (Xixin) 6.0 g, Dioscorea Rhizome (Shanyao) 12.0 g, Immature Orange Fruit (Zhishi) 6.0 g, Dried Tangerine Peel (Chenpi) 6.0 g, Pogostemon Herb (Huoxiang) 9.0 g
			—
			Japanese modification fomula
			JP Ephedra Herb 3.0 g, JP Glycyrrhiza 2.0 g, JP Apricot Kernel 3.0 g, JP Gypsum 10.0 g, JP Cinnamon Bark 3.0 g, JP Alisma Tuber 3.0 g, JP Poluporus Sclerotium 3.0 g, JP Atractylodes Rhizome 3.0 g, JP Poria Sclerotium 5.0 g, JP Bupleurum Root 5.3 g, JP Scutellaria Root 2.0 g, JP Pinellia Tuber 3.0 g, JP Ginger 3.0 g, Aster Root 3.0 g, Coltsfoot Flower 3.0 g, Blackberrylily Rhizome 3.0 g, JP Asiasarum Root 2.0 g, JP Discorea Rhizome 4.0 g, JP Immature Orange 2.0 g, JP Citrus Unshiu Peel 2.0 g, JP Pogostemon Herb 3.0 g
Shosaikoto	小柴胡湯	Xiao chai hu tang	JP Bupleurum Root 7.0 g, JP Pinellia Tuber 5.0 g, JP Scutellaria Root 3.0 g, JP Jujube 3.0 g, JP Ginseng 3.0 g, JP Glycyrrhiza 2.0 g, JP Ginger 1.0 g
Shosaikotokakikyosekko	小柴胡湯加桔梗石膏	Xiao chai hu tang jia jie geng shi gao	JP Gypsum 10.0 g, JP Bupleurum Root 7.0 g, JP Pinellia Tuber 5.0 g, JP Scutellaria Root 3.0 g, JP Platycodon Root 3.0 g, JP Jujube 3.0 g, JP Ginseng 3.0 g, JP Glycyrrhiza 2.0 g, JP Ginger 1.0 g

Niitsuma et al. reported two cases (case 1 and 2) of COVID-19 pneumonia (Niitsuma et al., 2020). In case 1, a 78-year-old male patient with a history of stroke and hypertension presented with complains of fever, dyspnea, malaise, and loss of appetite with a decreased oxygen saturation. Computed tomography (CT) revealed bilateral interstitial shadow, and a diagnosis of moderate stage II COVID-19 was made. The patient was administered Western medication including lopinavir/ritonavir, hydrocortisone, ceftriaxone, sulbactam/ampicillin, and acetaminophen. For the fever, cough, and sputum, Kampo medicine maoto, daiseiryuto, and chikujountanto were also sequentially administered. This multidisciplinary treatment approach relieved symptoms within 10 days. Case 2 was a 74-year-old woman with complains of joint pain, fever, and loss of appetite. CT revealed interstitial shadow in the left lung, and a diagnosis of moderate stage I COVID-19 was made. Additionally, the patient was administered Western medication including baloxavir marboxil, moxifloxacin, lopinavir/ritonavir, and warfarin. Kampo medicine maoto, followed by keishito and eppikajutsuto were also administered. This multidisciplinary approach relieved patient symptoms within 10 days.

Kashima et al. reported two cases of COVID-19 in which Kampo medicine may have contributed toward the suppression of a severe stage (cases 3 and 4) (Kashima et al., 2020). Case 3 was a 50-year-old woman with complains of fever, malaise, headache, blocked nose, lumbago, and loss of appetite. CT showed interstitial shadow in the right lung, and a diagnosis of moderate stage I COVID-19 was made. Western medicines such as acetaminophen and ciclesonide were prescribed. Kampo medicine kakkonto and shosaikoto, followed by shosaikoto and bukuryoingohangekobokuto were administered. All symptoms alleviated within 4 days. Whereas case 4 was a 53-year-old woman with systemic lupus erythematosus, who presented with cold and heat sensation, malaise, cough, lumbago, appetite loss, and diarrhea. CT showed interstitial shadow in the bilateral lung with a decrease in oxygen saturation, and a diagnosis of moderate stage II COVID-19 was made. Western medications such as ceftriaxone, azithromycin, acetaminophen, ciclesonide, and favipiravir were administered to the patient. Kampo medicine shosaikoto, saikanto, chikujountanto, bukuryoingohangekobokuto, jinsoin, hochuekkito, gokoto, kikyosekko, and keishibukuryogan were also prescribed according to the patients symptoms and condition. Finally, the patient recovered 20 days after admission.

Homma et al. reported a case of COVID-19 pneumonia treated with bakumondoto and hangekobokuto combined with Western medicine (case 5) (Homma et al., 2020b). Case 5 was a 30-year-old man with atopic dermatitis who presented with complains of headache, high fever, throat pain, cold cough, and malaise. CT showed interstitial shadow in the bilateral lungs with a decrease in oxygen saturation, which resulted in the diagnosis of moderate stage II COVID-19. Kampo medicine bakumondoto and hangekobokuto for cough were administered, following which all symptoms alleviated within 7 days. They also reported a case of pneumonia with facial paralysis and olfactory disturbance treated by maoto combined with Western medicine (case 6) (Homma et al., 2020a). Case 6 was a 35-year-old woman with complains of cough, malaise, sore throat, nausea, fever, headache, anosmia, dysgeusia, and facial paralysis. CT showed multiple ground-glass opacities in both the lungs which was diagnosed as moderate stage I COVID-19. Western medication acetaminophen, ciclesonide, and favipiravir were prescribed. Kampo medicine maoto was also used for respiratory and neural symptoms. The patients symptoms improved 11 days after admission.

Irie et al. reported three mild cases of COVID-19 treated with saikatsugekito (cases 7, 8, and 9) (Irie et al., 2020). All three cases recovered after treatment with Kampo medicine alone. Case 7 was a mild stage COVID-19 patient who was a 41-year-old woman with complains of headache, sore throat, nausea, taste disorder, and fever. She was treated with kakkonto and shosaikotokakikyosekko, and her condition improved within 10 days. While case 8 was a mild stage COVID-19 patient, a 16-year-old woman who presented with nasal congestion and taste disorder. She was treated with kakkonto and shosaikotokakikyosekko, and the patient’s condition improved within 4 days. Case 9 was a mild stage COVID-19 patient, a 12-year-old girl with complains of nasal congestion and taste disorder. She was also treated with kakkonto and shosaikotokakikyosekko, and the patient’s condition improved within 3 days.

Watanabe et al. reported two cases of COVID-19 with symptoms similar to a common cold, treated using modified Qing Fei Pai Du Tang (QFPDT) followed by hochuekkito under treatment with Western medicine (cases 10 and 11) (Watanabe and Watanabe, 2020). Case 10 was a 59-year-old man with complains of fever, sore through, anosmia, and abdominal fullness. The patient was administered Western medication including acetaminophen, clarithromycin, tranexamic acid, and L-carbocisteine, and administration of modified QFPDT for 1 week improved the patient’s symptoms. Following which, hochuekkito was administered to promote recovery. While case 11 was a 36-year-old woman with Hashimoto’s disease who presented with fever, cough, sputum, headache, and nasal bleeding. The patient was prescribed Western medication such as acetaminophen, clarithromycin, tranexamic acid, and L-carbocisteine. Following which, although she defervesced 1 week after the administration of modified QFPDT, complains of cough and sputum persisted. Kakkoshokisankagen were used thereafter, and symptoms such as cough, sputum, and malaise gradually improved. Finally, hochuekkito was administered to promote recovery.

Kyo reported three cases of pneumonia treated by a combination of Kampo medicine and Western medicine. These patients are presented as cases 12, 13, and 14 (Kyo 2020), and are a 40-year-old woman, a 64-year-old woman, and a 47-year-old man, respectively, with complains of high fever. They were diagnosed as having moderate stage II COVID-19. After the administration of Kampo formula, the patients defervesced within 3 days.

Yamasaki reported three cases of pneumonia treated by Kampo medicine in combination with Western medicine (cases 15 and 16) (Yamasaki 2020). Case 15 was a 67-year-old woman with a history of surgically resected lung cancer who complained of fever and malaise. CT showed infiltration shadow in the left lung, and the patient was diagnosed as having moderate stage I COVID-19. Administration of Kampo medicine combined with makyokansekito, ireito, and shosaikotokakikyosekko improved fever and malaise within 3 and 5 days, respectively. Case 16 was a 69-year-old man with type II diabetes mellitus with complains of fever and cough. CT showed infiltration shadow in the right lung that was diagnosed as moderate stage I COVID-19. Administration of Kampo medicine combined with makyokansekito, ireito, and shosaikotokakikyosekko improved fever and cough within 3 and 6 days, respectively.

Takayama et al. reported a case of pneumonia treated by Kampo medicine gokoto (case 17) (Takayama et al., 2021b). A 52-year-old male patient presented with cough, sputum, and joint pain. Chest radiography revealed interstitial shadow in the right lung that was diagnosed as moderate stage I COVID-19. After administration of gokoto, a Kampo medicine for cough and sputum, for symptom relief, cough and sputum improved within 4 days. Takayama et al. also reported 5 cases (cases 18–22) of COVID-19-related olfactory disorder treated by kakkontokasenkyushin’i (Takayama et al., 2021a). The symptoms improved within 3–5 days after administration of kakkontokasenkyushin’i. Kakkontokasenkyushin'i can be used for treating nasal congestion, rhinitis, and inflammation in the nasal mucosa. Olfactory disorder in COVID-19 has been reported to be associated with inflammation and congestion, especially in the olfactory bulb and olfactory cleft. They concluded that kakkontokasenkyushin’i may be considered as a treatment alternative for the olfactory disorder related to COVID-19.

On the other hand, some reports have suggested the clinical efficacy of Chinese medicine in COVID-19. Ke et al. have reported the efficacy of Lianhua Qingwen (LH) capsules in patients with COVID-19, using a multi-center, prospective, randomized controlled trial (Hu et al., 2020). Certain LH components overlap in the Kampo medicine maoto or maotokasekko that were administered to cases 1–4, 6–11, and 14–22, as shown in Tables 1, 2. The efficacy of LH was compared between conventional treatment alone or in combination with LH for 14 days. The rate of recovery of symptoms including fever, fatigue, and cough was significantly shortened by LH administration. Zheng et al. reported that LH treatment modulates the inflammatory process, exerts antiviral effects and repairs lung injury in the network pharmacology analysis of the therapeutic mechanisms of LH in COVID-19 (Zheng et al., 2020).

Furthermore, a national retrospective registry study reported by Lihua et al. suggested that QFPDT was associated with a substantially lower risk of in-hospital mortality (Zhang et al., 2021). Modified QFPDT in Japan was administered to cases 10, 11, 15, and 16, as shown in Tables 1, 2. Although these reports have revealed the efficacy of traditional medicine, further clinical trials are required to evaluate the efficacy of Kampo medicine on COVID-19-related symptoms and conditions.

## JSOM Research Project on the Use of Kampo Medicine in Treating COVID-19

The JSOM has prepared a research project for clinical trials of Kampo medicine in patients with COVID-19 (Takayama et al., 2020a; Takayama et al., 2020c; Namiki et al., 2021), some of which are currently in progress. Table 3 shows the list of the clinical studies being planned or conducted in the JSOM projects. In particular, studies one through three are referred to as an Integrative Management in Japan for Epidemic Disease (IMJEDI).

**TABLE 3:** Outline of ongoing research on the prevention and treatment for COVID-19 in Kampo medicine.

Study of JSOM research	Trial design	Subjects	Number of subjects	Intervention	Comparison	Outcome	Aim	Registration
Study 1 (Prevention)	A multi-center, interventional, parallel-group, randomized (1:1 ratio), investigator-sponsored, two-arm study	Healthy hospital workers	Set at 6,000	Participants receive hochuekkito in 9 tablets 2 times per day for 8 weeks	Participants receive placebo in the same dosage as the intervention group	Primary outcomes: Number of patients with a SARS-CoV-2 RNA by polymerase chain reaction (PCR) positive result with at least one symptom (fever, cough, sputum, malaise, shortness of breath) during the 12 weeks study period (including the 4 weeks observation period after oral administration)	To test our hypothesis that hochuekkito has a preventive effect on the symptoms of COVID-19	jRCTs031200150 registered on October 14, 2020
Study 2 (Acute treatment)	Multicenter, retrospective observational study	Mild to moderate COVID-19 patients treated with conventional medicines/Kampo medicines	Set at 1,000	N/A	N/A	Primary outcomes: Treatment, symptom course, critical illness outcome	To investigate the efficacy of the actual treatment (the efficacy of conventional and Kampo medicines) in patients with mild to moderate or suspected COVID-19	UMIN000041301 registered on August 4, 2020
Study 3 (acute treatment)	A multi-center, interventional, parallel-group, randomized (1:1 ratio), investigator-sponsored, two-arm study	Mild to moderate COVID-19 patients	Set at 150	Patients will receive 2.5 g of KT (TJ-1@TSUMURA and Co.) and 2.5 g of SSKKS (TJ-109@TSUMURA and Co.) 3 times a day, orally, for 14 days in addition to the conventional treatment	Patients will receive conventional treatment	Primary outcomes: The number of days till at least one of the symptoms (fever, cough, sputum, malaise, shortness of breath) improves in the first 14 days of treatment	To test our hypothesis that additional administration of kakkonto and shosaikotokakikyosekko is more effective in relieving symptoms and preventing the onset of severe infection in mild-to-moderate COVID-19 patients compared to treatment with only conventional treatment	jRCTs021200020. registered on August 25, 2020
Study 4 (Sequelae treatment)	Multicenter, prospective observational study	Patients with COVID-19 related sequelae	N/A	N/A	N/A	Outcomes: visual analogue scale (VAS) in each symptom (fatigue, short of breathing, joint pain, chest pain, cough, dysgeusia, anosmia, etc), SF-12 (evaluation scale for health related quality of life), five-grade evaluation in overall treatment efficacy, safety evaluation	To investigate the efficacy and safety of Kampo treatment in patients with COVID-19 related sequelae	UMIN000044318 registered on May 25, 2021

Notes: University Hospital Medical Information Network; UMIN.

Japan Registry of Clinical Trials; jRCT.

### Prevention (Pre-Disease)

Study 1 is a multi-centered, randomized trial to test our hypothesis that the Kampo medicine, hochuekkito, has a preventive effect on the symptoms of COVID-19 among healthy hospital workers (Namiki et al., 2021). While we hope for an effective and widely available vaccine, the efficacy of the current vaccines has not been shown in a large number of patients. Furthermore, the efficacy of the vaccines may be reduced if SARS-CoV-2 persistently and consistently undergoes mutations (Awadasseid et al., 2021; Lauring and Hodcroft 2021). The possible mechanisms of the preventive effect of hochuekkito (bu Zhong yi qi tang) against COVID-19 have reported by Takayama et al. (Takayama et al., 2020b). Thus, clinical trials are being conducted to assess the efficacy of Kampo medicine for preventing COVID-19. Medical staff, overextended from the increase in critical patient care and the other circumstances involved with the COVID-19 pandemic, are thought to have decreased immunity along with physical and mental health issues. Furthermore, they are at an increased risk of infection when providing medical care. Therefore, we considered it necessary to verify the effectiveness of Kampo medicine in preventing illness among staff members who continue to provide medical care despite these challenges.

### Treatment (Especially in the Acute and Subacute Stages of Disease)

Study 2 is a multi-centered, retrospective, observational study to investigate the efficacy of the actual treatment (conventional and Kampo medicine) in patients with mild-to-moderate or even suspected COVID-19 (Takayama et al., 2020a). Several clinical trials on antiviral drugs are currently in progress; however, evidence on its efficacy remains limited. Kampo medicine has a long history of repeated use in viral epidemics and was also used during the Spanish influenza pandemic, approximately 100 years ago. Several cases of COVID-19 treated with Kampo medicine have already been reported (Homma et al., 2020a; Homma et al., 2020b; Irie et al., 2020; Kashima et al., 2020; Kyo 2020; Niitsuma et al., 2020; Watanabe and Watanabe, 2020; Yamasaki 2020; Takayama et al., 2021a; Takayama et al., 2021b). The next step in establishing Kampo medicine as a treatment strategy for COVID-19 is organizing a clinical study with a large number of cases. In Japan, many COVID-19 patients are treated with a combination of Kampo medicine and Western medicine, and an observational study that would collect data from a multi-center setting would be ideal.

Study 3 is a multi-centered, randomized trial to test our hypothesis that additional administration of Kampo medicines kakkonto and shosaikotokakikyosekko is more effective in relieving symptoms and preventing the onset of severe infection in mild-to-moderate COVID-19 patients compared to those treated with conventional treatment alone (Takayama et al., 2020c). The symptoms and signs associated with acute infectious diseases were categorized into six-stage patterns in the original concept of Kampo medicine described in the Shanhanlun, which is a traditional Asian medical textbook of infectious diseases written by Zhan Zhongjing (Takayama et al., 2017; The Dictionary of Kampo Medicine, 2020). The Shanhanlun explains that infectious diseases progress through six-stage patterns: early, middle, and late yang patterns and early, middle, and late yin patterns. Characteristic symptoms are chill and fever in the early yang pattern, alternating chill and fever with respiratory and intestinal symptoms in the middle yang pattern, and marked fever with thirst in the late yang pattern. The symptoms also include coldness with intestinal symptoms in the early yin pattern, coldness with marked fatigue in the middle yang pattern, and coldness with circulation failure with disturbance of consciousness in the late yang pattern. To study the efficacy and safety of Kampo medicines for COVID-19 patients, it is necessary to focus on a certain type of Kampo medicine. COVID-19 symptoms include chills, fever, muscle pain, joint pain, nasal congestion and discharge, sore throat, a cough producing sputum, loss of appetite, diarrhea, impaired smell and taste, and malaise, which progress from the early yang pattern to the middle and late yang patterns described in Shanhanlun. The type of Kampo formula depends on the stage of clinical manifestation: mao formula in the early yang pattern, saiko formula in the middle yang pattern, and sekko in the late yang pattern. A combination of mao formula, saiko formula, and sekko is an ideal choice for treating diseases such as COVID-19, which transition from the early to the middle and late yang patterns in the Kampo concept. Thus, these combinations were included in saikatsugekito, which was used for the Spanish flu, and case series have already reported the use of saikatsugekito for COVID-19 (Irie et al., 2020). Saikatsugekitois applied in combination with kakkonto and shosaikotokakikyosekko under the permissions of the national health insurance. The possible mechanisms of saikatsugekito against SARS-CoV-2 and COVID-19 have been reported by Arita et al. (Arita et al., 2020).

### Recovery and Sequelae (Post-Disease)

While study 4 is a multi-centered, prospective, observational study to investigate the efficacy of Kampo medicines in patients with COVID-19-related sequelae. After the stabilization of COVID-19 patients, continued support for recovery and management of sequelae is needed. Future research interests include epidemiological investigations of sequelae and case series that summarize the experience of patients who used Kampo medicine for treatment.

## Discussion

### Prospects of a Clinical Trial of Kampo Medicine for COVID-19

The contribution and prospects of the present projects on Kampo medicine and its efficacy as a COVID-19 treatment are as follows:

• Kampo medicine may act on viruses, human tissues, and organs via multiple mechanisms that differ from those of other drugs being developed in Western medicine (Arita et al., 2020; Takayama et al., 2020b).
• Kampo medicines for common cold are well established; their possible side effects are generally known and safety information has already been acquired (AuthorAnonymous, 2005; Kubo et al., 2013; Shimamoto et al., 2014; Takayama et al., 2017; Arai et al., 2018; Takayama et al., 2018; Takayama et al., 2020d).
• Kampo medicines are inexpensive, and if the clinical studies show their efficacy, it would be possible to provide care to many health care workers and patients, thus reducing medical expenses and providing medical economic benefits (Arita et al., 2015).
• If Kampo medicine is shown to be effective in alleviating symptoms and reducing the severity of disease, it could help to address the depletion of medical resources. There are currently no reports on effective treatments for the sequelae of COVID-19; if a clinical study could help develop a treatment strategy for sequelae, this could benefit many patients with such issues.
• Since viral mutations are now rapidly progressing, Kampo medicine could serve as an option in cases of resistance to Western medicine (Awadasseid et al., 2021; Lauring and Hodcroft 2021).

The quality control of Kampo medicines in Japan is highly regarded internationally, with very low variability from product to product. In this respect, conducting clinical research using Japanese Kampo medicines also has the advantage of maintaining uniformity of treatment for consistency and accuracy of research results.

### Attention to Use Qing Fei Pai Du Tang From China

QFPDT is recommended for use in patients as an option for the treatment of COVID-19 in the seventh edition of the Chinese COVID-19 guidelines (National Health Commission and National Administration of Traditional Chinese Medicine 2020). In an observational study, 98 patients were treated with QFPDT for 9 days, and the efficiency of relief in major symptoms was 91.6% (Wang et al., 2020). However, randomized controlled trials of QFPDT have never been reported previously. Furthermore, QFPDT includes crude drugs which are not approved by the regulatory authorities in Japan. Thus, it is difficult to reproduce and use the same products in Japan. On the other hand, it is possible to reproduce similar Kampo medicines by combining certain Kampo extract preparations such as gokoto and saireito in Japan. The original QFPDT includes 21 crude drugs; however, their dosages are much higher than the usual dosage in Japan. The JSOM reminds us that the duration of administration should be evaluated every 3 days for a maximum of 2 weeks. It is also important to note that while the drug is indicated for use in young people, it should be used with sufficient caution in the elderly (The Japan Society for Oriental Medicine, 2021).

### Side Effects of Kampo Medicine

Guidelines for the management of hypertension (2014) introduced the side effect associated with glycyrrhizin, which has the potential to induce pseudo-aldosteronism (Shimamoto et al., 2014). A consensus statement for the diagnosis and treatment of drug-induced lung injuries (Kubo et al., 2013) reported the side effect of interstitial pneumonia with the use of shosaikoto (AuthorAnonymous, 2005). The prevalence of shosaikoto-induced interstitial pneumonia is reported to be under 0.1% (Arai et al., 2018); however, when limited in patients with no prior sensitization to shosaikoto, there were no reports of new-onset shosaikoto-induced interstitial pneumonia within 2 weeks after its administration. Ephedrae Herba also includes ephedrine, which induces an adrenergic reaction. Thus, several clinical guidelines recommend that this medicine should be cautiously prescribed by the Mao and Saiko formula in those with hypokalemia, heart disease, and prior sensitization of allergy to shosaikoto (Arita et al., 2015; Takayama and Iwasaki, 2017; Takayama et al., 2020d).

## Conclusion

Several cases treated with Kampo medicine have reported in COVID-19. The JSOM research will enable us to propose a wide range of treatments for emerging viral infections, i.e., not only for COVID-19 but also for viruses we may encounter in the future.

## References

[B1] Arai I. Hagiwara Y. Motoo Y. (2018). Estimated Incidence of Adverse Reactions to Kampo Medicines in Randomized Controlled Clinical Trials. Traditional Kampo Med. 5, 106–112. 10.1002/tkm2.1200

[B2] Arita R. Ono R. Saito N. Takayama S. Namiki T. Ito T. (2020). Kakkonto, Shosaikoto, Platycodon Grandiflorum Root, and gypsum (A Japanese Original Combination Drug Known as Saikatsugekito): Pharmacological Review of its Activity against Viral Infections and Respiratory Inflammatory Conditions and a Discussion of its Applications to COVID ‐19. Traditional Kampo Med. 7, 115–127. 10.1002/tkm2.1258

[B3] Arita R. Yoshino T. Hotta Y. Miyamoto Y. Osawa I. Takemoto O. (2015). National Cost Estimation of Maoto, a Kampo Medicine, Compared with Oseltamivir for the Treatment of Influenza in Japan. Traditional Kampo Med. 3, 59–62. 10.1002/tkm2.1027

[B5] Awadasseid A. Wu Y. Tanaka Y. Zhang W. (2021). SARS-CoV-2 Variants Evolved during the Early Stage of the Pandemic and Effects of Mutations on Adaptation in Wuhan Populations. Int. J. Biol. Sci. 17, 97–106. 10.7150/ijbs.47827 33390836 PMC7757051

[B6] Carfì A. Bernabei R. Landi F. (2020). Persistent Symptoms in Patients after Acute COVID-19. JAMA 324, 603–605. 10.1001/jama.2020.12603 32644129 PMC7349096

[B7] Ferretti L. Wymant C. Kendall M. Zhao L. Nurtay A. Abeler-Dörner L. (2020). Quantifying SARS-CoV-2 Transmission Suggests Epidemic Control with Digital Contact Tracing. Science 368, eabb6936. 10.1126/science.abb6936 32234805 PMC7164555

[B4] Goto S. Lauda D. Katai S. Yasui H. (2005). “Appendix - Composition and Indications of 148 Prescriptions,” Current Kampo Medicine. Berkeley: International Institute of Health and Human Services, 85–101.1559-033X

[B8] He X. Lau E. H. Y. Wu P. Deng X. Wang J. Hao X. (2020). Temporal Dynamics in Viral Shedding and Transmissibility of COVID-19. Nat. Med. 26, 672–675. 10.1038/s41591-020-0869-5 32296168

[B9] Hoffmann M. Kleine-Weber H. Schroeder S. Krüger N. Herrler T. Erichsen S. (2020). SARS-CoV-2 Cell Entry Depends on ACE2 and TMPRSS2 and Is Blocked by a Clinically Proven Protease Inhibitor. Cell 181, 271–e8. 10.1016/j.cell.2020.02.052 32142651 PMC7102627

[B10] Homma Y. Inoue K. Moritaka T. (2020a). A Case of New Coronavirus (SARS-CoV-2) Pneumonia that Required Oxygen Administration but Improved with Symptomatic Treatment Alone. Infect. Dis, 1–2. (in Japanese). Available at: https://www.kansensho.or.jp/uploads/files/topics/2019ncov/covid19_casereport_200422_2.pdf.

[B11] Homma Y. Watanabe M. Inoue K. Moritaka T. (2020b). Coronavirus Disease-19 Pneumonia with Facial Nerve Palsy and Olfactory Disturbance. Intern. Med. 59, 1773–1775. 10.2169/internalmedicine.5014-20 32669517 PMC7434541

[B12] Hu K. Guan W. J. Bi Y. Zhang W. Li L. Zhang B. (2020). Efficacy and Safety of Lianhuaqingwen Capsules, a Repurposed Chinese Herb, in Patients with Coronavirus Disease 2019: A Multicenter, Prospective, Randomized Controlled Trial. Phytomedicine 85, 153242. 10.1016/j.phymed.2020.153242 33867046 PMC7229744

[B13] Influenza (2008). Department of Health, Ministry of Home Affairs in Japan. Tokyo, Japan: Heibonsha.

[B14] Irie Y. Nakae S. (2020). The Role of Kampo Medicines in the Pandemic of Viral Infections: Learning from the Spanish Flu. Kampo Med. 71, 272–283.

[B15] Irie Y. Nakae H. Fukui S. (2020). Three Mild Cases of Coronavirus Disease 2019 Treated with Saikatsugekito, a Japanese Herbal Medicine. Traditional Kampo Med. 8, 111–114. 10.1002/tkm2.1261

[B16] Jarvis M. C. (2020). Aerosol Transmission of SARS-CoV-2: Physical Principles and Implications. Front. Public Health 8, 590041. 10.3389/fpubh.2020.590041 33330334 PMC7719704

[B17] Kashima M. Hayano S. Iwagoe H. (2020). Two Cases of COVID-19 in Which Kampo Medicine May Have Contributed to the Suppression for Severe Stage. Home page of the Japanese Association for. Infect. Dis, 1–5. (in Japanese). Available at: https://www.kansensho.or.jp/uploads/files/topics/2019ncov/covid19_casereport_200430_3.pdf.

[B18] Kubo K. Azuma A. Kanazawa M. Kameda H. Kusumoto M. Genma A. (2013). Consensus Statement for the Diagnosis and Treatment of Drug-Induced Lung Injuries. Respir. Investig. 51, 260–277. 10.1016/j.resinv.2013.09.001 24238235

[B19] Kyo S. (2020). Thirty Nine Case Reports of Kampo Treatment in COVID-19. J. Kampo Med. 67, 953–963.

[B20] Lauring A. S. Hodcroft E. B. (2021). Genetic Variants of SARS-CoV-2—What Do They Mean?. JAMA 325, 529. 10.1001/jama.2020.27124 33404586

[B21] Lu R. Zhao X. Li J. Niu P. Yang B. Wu H. (2020). Genomic Characterisation and Epidemiology of 2019 Novel Coronavirus: Implications for Virus Origins and Receptor Binding. Lancet 395, 565–574. 10.1016/S0140-6736(20)30251-8 32007145 PMC7159086

[B22] McElvaney O. J. McEvoy N. L. McElvaney O. F. Carroll T. P. Murphy M. P. Dunlea D. M. (2020). Characterization of the Inflammatory Response to Severe COVID-19 Illness. Am. J. Respir. Crit. Care Med. 202 (6), 812–821. 10.1164/rccm.202005-1583OC 32584597 PMC7491404

[B23] Ministry of Health, Labour and Welfare of Japan (2020a). A Basic Guidelines for Infection Control against COVID-19 25 February 2020. [cited 7 Jan 2021] Available at: https://www.mhlw.go.jp/content/10900000/000599698.pdf.

[B24] Ministry of Health, Labour and Welfare of Japan (2020b). COVID-19 Guide to Medical Treatment 2020, Version 2.2. 2020. (in Japanese) Available at: https://www.mhlw.go.jp/content/000646531.pdf.

[B25] Ministry of Health, Labour and Welfare of Japan (2020c). COVID-19 Guide to Medical Treatment 2020, Version 4.1, P29. (in Japanese) Available at: https://www.mhlw.go.jp/content/000712473.pdf.

[B26] Nabeshima S. Kashiwagi K. Ajisaka K. Masui S. Takeoka H. Ikematsu H. (2012). A Randomized, Controlled Trial Comparing Traditional Herbal Medicine and Neuraminidase Inhibitors in the Treatment of Seasonal Influenza. J. Infect. Chemother. 18, 534–543. 10.1007/s10156-012-0378-7 22350323

[B27] Namiki T. Takayama S. Arita R. Ishii T. Kainuma M. Makino T. (2021). A Structured Summary of a Study Protocol for a multi-center, Randomized Controlled Trial (RCT) of COVID-19 Prevention with Kampo Medicines (Integrative Management in Japan for Epidemic Disease by Prophylactic Study: IMJEDI P1 Study). Trials 22, 23. 10.1186/s13063-020-04939-2 33407828 PMC7787232

[B28] National Institute of Infectious Diseases (2020). COVID-19. Infectious Agents Surveillance Report 41. Japan: National Institute of Infectious Diseases, 103–105. Available at: https://www.niid.go.jp/niid/images/idsc/iasr/41/485.pdf.

[B29] Niitsuma K. Suzuki T. Saito M. (2020). Two Cases of Novel Coronavirus (COVID-19) Pneumonia that Had Occurred Asymptomatically Including a Severe Case of Organized Pneumonia Pattern. Home page of the Japanese Association for. Infect. Dis , 1–5. (in Japanese). Available at: https://www.kansensho.or.jp/uploads/files/topics/2019ncov/covid19_casereport_200331_3.pdf.

[B30] Shimamoto K. Ando K. Fujita T. Hasebe N. Higaki J. Horiuchi M. (2014). The Japanese Society of Hypertension Guidelines for the Management of Hypertension (JSH 2014). Hypertens. Res. 37, 253–390. 10.1038/hr.2014.20 24705419

[B32] Takayama S. Arita R. Iwasaki K. (2017). How Do You TreatUpper Respiratory Infectionsin the Elderly in Your Practice? Med. Acupuncture 29, 105–113. 10.1089/acu.2017.29047.cpl

[B33] Takayama S. Arita R. Kikuchi A. Ohsawa M. Kaneko S. Ishii T. (2018). Clinical Practice Guidelines and Evidence for the Efficacy of Traditional Japanese Herbal Medicine (Kampo) in Treating Geriatric Patients. Front. Nutr. 5, 66. 10.3389/fnut.2018.00066 30083536 PMC6064728

[B34] Takayama S. Arita R. Ono R. Saito N. Suzuki S. Kikuchi A. (2021a). Treatment of COVID-19-Related Olfactory Disorder Promoted by Kakkontokasenkyushin'i: a Case Series. Tohoku J. Exp. Med 254, 71–80. 10.1620/tjem.254.71 34108344

[B35] Takayama S. Iwasaki K. (2017). Systematic Review of Traditional Chinese Medicine for Geriatrics. Geriatr. Gerontol. Int. 17, 679–688. 10.1111/ggi.12803 27273639

[B36] Takayama S. Kashima M. Namiki T. Ito T. Ono R. Arita R. (2020a). Conventional and Kampo Medicine in the Treatment of Mild to Moderate COVID-19: A Multicenter, Retrospective Observational Study Protocol by the Integrative Management in Japan for Epidemic Disease (IMJEDI Study-Observation). Tradit Kampo Med. 8, 106–110. 10.1002/tkm2.1271

[B37] Takayama S. Kikuchi A. Makino T. Kainuma M. Namiki T. Ito T. (2020b). Basic Pharmacological Mechanisms and Clinical Evidence of the Efficacy of Hochuekkito against Infectious Diseases and its Potential for Use against COVID‐19. Tradit Kampo Med. 8, 3–21. 10.1002/tkm2.1264

[B38] Takayama S. Namiki T. Ito T. Arita R. Nakae H. Kobayashi S. (2020c). A multi-center, Randomized Controlled Trial by the Integrative Management in Japan for Epidemic Disease (IMJEDI Study-RCT) on the Use of Kampo Medicine, Kakkonto with Shosaikotokakikyosekko, in Mild-To-Moderate COVID-19 Patients for Symptomatic Relief and Prevention of Severe Stage: a Structured Summary of a Study Protocol for a Randomized Controlled Trial. Trials 21, 827. 10.1186/s13063-020-04746-9 33008479 PMC7530547

[B39] Takayama S. Ono R. Aria R. Saito N. Suzuki S. Tadano Y. (2021b). Usefulness of Portable Chest Radiography and Blood Sampling for Prompt Medical Response in COVID-19 Isolation Facilities: Two Cases of Moderate Stage I COVID-19. J. Hosp. Gen. Med. 3(3), 92–96.

[B40] Takayama S. Tomita N. Arita R. Ono R. Kikuchi A. Ishii T. (2020d). Kampo Medicine for Various Aging-Related Symptoms: A Review of Geriatric Syndrome. Front. Nutr. 7, 86. 10.3389/fnut.2020.00086 32766269 PMC7381143

[B42] The Dictionary of Kampo Medicine (2020). The Japan Society for Oriental Medicine. Kyoto: Medical Yukon Publishing Co., Ltd..

[B43] The Japan Society for Oriental Medicine (2021). Notice of Alert on the Use of a New Herbal Medicine Product from China. Tokyo: Home page of The Japan Society for Oriental Medicine. [cited 7 Jan 2021]. Available at: http://www.jsom.or.jp/medical/notice/pdf/covid-attention-medical.pdf.

[B44] Wang R. Sijin Y. Chunguang X. Qilin S. Minqing L. Liao L. (2020). Clinical Efficacy of Qing Lung Detoxification in the Treatment of Novel Coronavirus Pneumonia. Beijing: Pharmacology and Clinics of Chinese Materia Medica; (in Chinese). 10.13412/j.cnki.zyyl.20200303.002

[B45] Watanabe K. Watanabe N. (2020). Experience in the Treatment of COVID-19 with Qingfei Paidu Decoction. J. Kampo Med. 67, 785–790.

[B46] Wei P-F. (2020). Diagnosis and Treatment Protocol for Novel Coronavirus Pneumonia (Trial Version 7). Chin. Med. J. (Engl) 133 (9), 1087–1095. 10.1097/CM9.0000000000000819 32358325 PMC7213636

[B47] World Health Organization (2019). Novel Coronavirus (2019-nCOV) SITUATION REPORT-1. 21 January 2020. [cited 7 Jan 2021]. Available at: https://www.who.int/docs/default-source/coronaviruse/situation-reports/20200121-sitrep-1-2019-ncov.pdf.

[B48] World Health Organization (2015). Summary of Probable SARS Cases with Onset of Illness from 1 November 2002 to 31 July 2003. [cited 7 Jan 2021]. Available at: https://www.who.int/publications/m/item/summary-of-probable-sars-cases-with-onset-of-illness-from-1-november-2002-to-31-july-2003.

[B49] World Health Organization (2020). WHO Director-General’s Opening Remarks at the media Briefing on COVID-19 – 11 March 2020. [cited 7 Jan 2021]. Available at: https://www.who.int/director-general/speeches/detail/who-director-general-s-opening-remarks-at-the-media-briefing-on-covid-19---11-march-2020.

[B50] Wu Z. McGoogan J. M. (2020). Characteristics of and Important Lessons from the Coronavirus Disease 2019 (COVID-19) Outbreak in China: Summary of a Report of 72 314 Cases from the Chinese Center for Disease Control and Prevention (COVID-19) Outbreak in China: Summary of a Report of 72,314 Cases from the Chinese Center for Disease Control and Prevention. JAMA 323, 1239–1242. 10.1001/jama.2020.2648 32091533

[B51] Yamasaki G. (2020). Two Cases of COVID-19 Treated with Makyokansekitogoireitogo and Shosaikotokakikyosekko. J. Kampo Med. 67, 933–998.

[B52] Yasui H. (2007). History of the Schools of Kampo Medicine. Kampo Med. 58, 177–202. 10.3937/kampomed.58.177 Available at: https://www.jstage.jst.go.jp/article/kampomed/58/2/58_2_177/_pdf/-char/ja.

[B53] Yoshino T. Arita R. Horiba Y. Watanabe K. (2019). The Use of Maoto (Ma-Huang-Tang), a Traditional Japanese Kampo Medicine, to Alleviate Flu Symptoms: a Systematic Review and Meta-Analysis. BMC. Complement. Altern. Med. 19, 68. 10.1186/s12906-019-2474-z 30885188 PMC6421694

[B54] Zaki A. M. van Boheemen S. Bestebroer T. M. Osterhaus A. D. Fouchier R. A. (2012). Isolation of a Novel Coronavirus from a Man with Pneumonia in Saudi Arabia. N. Engl. J. Med. 367, 1814–1820. 10.1056/NEJMoa1211721 23075143

[B55] Zhang L. Zheng X. Bai X. Wang Q. Chen B. Wang H. (2021). Association between Use of Qingfei Paidu Tang and Mortality in Hospitalized Patients with COVID-19: A National Retrospective Registry Study. Phytomedicine 85, 153531. 10.1016/j.phymed.2021.153531 33799224 PMC7914374

[B56] Zheng S. Baak J. P. Li S. Xiao W. Ren H. Yang H. (2020). Network Pharmacology Analysis of the Therapeutic Mechanisms of the Traditional Chinese Herbal Formula Lian Hua Qing Wen in Corona Virus Disease 2019 (COVID-19), Gives Fundamental Support to the Clinical Use of LHQW. Phytomedicine 79, 153336. 10.1016/j.phymed.2020.153336 32949888 PMC7474845

[B57] Zhou P. Yang X. L. Wang X. G. Hu B. Zhang L. Zhang W. (2020). A Pneumonia Outbreak Associated with a New Coronavirus of Probable Bat Origin. Nature 579, 270–273. 10.1038/s41586-020-2012-7 32015507 PMC7095418

