# Supplementary material for: Prevention and Recovery of COVID-19 Patients With Kampo Medicine: Review of Case Reports and Ongoing Clinical Trials
Source: Front Pharmacol. 2021 Jun 23;12:656246. doi: 10.3389/fphar.2021.656246 (PMC8261067; doi:10.3389/fphar.2021.656246)
Supplement: Supplementary file 1 [file Table1.pdf]

Supplementary Table: Plant names and part of each ingredient

Ingredient in English (Chinese)	Plant name (Latin)	Plant part (Latin)
Alisma Tuber (Zexie)	*Alisma plantago-aquatica* Linné *(Alismataceae)*	*Tuber*
Apricot Seed (Xingren)	*Prunus armeniaca* Linné *(Rosaceae)*	*Semen*
Areca Pericarp	*Areca catechu* Linné *(Arecaceae)*	*Pericarpium*
Asiasarum Root and Rhizome (Xixin)	*Asiasarum sieboldii* F. Maekawa *(Aristolochiaceae)*	*Radix et Rhizoma*
Aster Root (Ziwan)	*Aster tataricus* Linné filius *(Compositae)*	*Radix*
Atractylodes Rhizome (Baizhu)	*Atractylodes macrocephala* Koidzumi (*Atractylodes ovata* De Candolle) *(Compositae)*	*Rhizoma*
Bamboo Culm	*Bambusa textilis* McClure, *Bambusa pervariabilis* McClure, *Bambusa beecheyana* Munro, *Bambusa tuldoides* Munro, *Phyllostachys nigra* Munro var. *henonis* Stapf ex Rendle, or *Phyllostachys bambusoides* Siebold et Zuccarini *(Gramineae)*	*Caulis*
Bitter Apricot Seed (Kuxingren)	*Prunus armeniaca* Linné *(Rosaceae)*	*Semen*
Blackberrylily Rhizome (Shegan)	*Belamcanda chinensis* Goldblatt et Mabb. (*Iris domestica* Linné) *(Iridaceae)*	*Rhizoma*
Bupleurum Root (Chaihu)	*Bupleurum chinense* DC *(Umbelliferae)*	*Radix*
Chinese Rhubarb Rhizome (Dahuang)	*Rheum palmatum* Linné *(Polygonaceae)*	*Rhizoma*
Cinnamon Twig (Guizhi)	*Cinnamomum cassia* Blume *(Lauraceae)*	*Ramulus*
Common Coltsfoot Flower (Kuandonghua)	*Tussilago farfara* Linné *(Compositae)*	*Flos*
Dioscorea Rhizome (Shanyao)	*Dioscorea polystachya* Turcz. *(Dioscoreaceae)*	*Rhizoma*
Dried Tangerine Peel (Chenpi)	*Citrus reticulata* Blanco *(Rutaceae)*	*Pericarpium*
Ephedra Herb (Mahuang)	*Ephedra sinica* Stapf *(Ephedraceae)*	*Herba*
Forsythia Fruit (Lianqiao)	*Forsythia suspensa* (Thunb.) Vahl. *(Oleaceae)*	*Fructus*
Ginger (Shengjiang)	*Zingiber officinale* Roscoe *(Zingiberaceae)*	*Rhizoma*
Glycyrrhiza Root (Gancao)	*Glycyrrhiza uralensis* Fischer *(Leguminosae)*	*Radix*
Glycyrrhiza Root and Rhisome processed with honey (Zhigancao)	*Glycyrrhiza uralensis* Fischer *(Leguminosae)*	*Radix et Rhizoma Praeparata cum Melle*
Gypsum (Shigao/Shengshigao)	*Gypsum Fibrosum*	-
Heartleaf Houttuynia Herb (Yuxingcao)	*Houttuynia cordata* Thunb. *(Saururaceae)*	*Herba*
Immature Orange Fruit (Zhishi)	Citrus trifoliata Linné (Rutaceae)	*Fructus immaturus*
Indigo Woad Root (Banlangen)	*Isatis tinctoria* Linné *(Brassicaceae)*	*Radix*
JP Alisma Tuber	*Alisma orientale* Juzepczuk *(Alismataceae)*	*Tuber*
JP Angelica Dahurica Root	*Angelica dahurica* Bentham et Hooker filius ex Franchet et Savatier *(Umbelliferae)*	*Radix*
JP Apricot Kernel	*Prunus armeniaca* Linné, *Prunus armeniaca* Linné *var. ansu* Maximowicz, or *Prunus sibirica* Linné *(Rosaceae)*	*Semen*
JP Asiasarum Root	*Asiasarum sieboldii* F. Maekawa, or *Asiasarum heterotropoides* F. Maekawa *var. mandshuricum* F. Maekawa *(Aristolochiaceae)*	*Radix*
JP Astragalus Root	*Astragalus membranaceus* Bunge *et Astragalus mongholicus* Bunge *(Leguminosae)*	*Radix*
JP Atractylodes Lancea Rhizome	*Atractylodes lancea* De Candolle, or *Atractylodes chinensis* Koidzumi *(Compositae)*	*Rhizoma*

JP Atractylodes Rhizome	*Atractylodes japonica* Koidzumi *ex* Kitamura, or *Atractylodes macrocephala* Koidzumi (*Atractylodes ovata* De Candolle) *(Compositae)*	*Rhizoma*
JP Brown Rice	*Oryza sativa* Linné *(Gramineae)*	*Fructus*
JP Bupleurum Root	*Bupleurum falcatum* Linné *(Umbelliferae)*	*Radix*
JP Chrysanthemum Flower	*Chrysanthemum morifolium* Ramatulle, or *Chrysanthemum indicum* Linné *(Compositae)*	*Flos*
JP Cimicifuga Rhizome	*Cimicifuga simplex* Turczaninow, *Cimicifuga dahurica* Maximowicz, *Cimicifuga foetida* Linné, or *Cimicifuga heracleifolia* Komarov *(Ranunculaceae)*	*Rhizoma*
JP Cinnamon Bark	*Cinnamomum cassia* Blume *(Lauraceae)*	*Cortex*
JP Citrus Unshiu Peel	*Citrus unshiu* Marcowicz, or *Citrus reticulata* Blanco *(Rutaceae)*	*Pericarpium*
JP Cnidium Rhizome	*Cnidium officinale* Makino *(Umbelliferae)*	*Rhizoma*
JP Coptis Rhizome	*Coptis japonica* Makino, *Coptis chinensis* Franchet, *Coptis deltoidea* C.Y. Cheng et Hsiao, or *Coptis teeta* Wallich *(Ranunculaceae)*	*Rhizoma*
JP Cyperus Rhizome	Cyperus rotundus Linné (Cyperaceae)	*Rhizoma*
JP Discorea Rhizome	Dioscorea japonica Thunberg, or Dioscorea batatas Decaisne (Dioscoreaceae)	*Rhizoma*
JP Ephedra Herb	*Ephedra sinica* Stapf, *Ephedra intermedia* Schrenk et C. A. Meyer, or *Ephedra equisetina* Bunge *(Ephedraceae)*	*Herba*
JP Forsythia Fruit	*Forsythia suspensa* Vahl *(Oleaceae)*	*Fructus*
JP Ginger	Zingiber officinale Roscoe *(Zingiberaceae)*	*Rhizoma*
JP Ginseng	Panax ginseng C. A. Meyer (Panax schinseng Nees) (Araliaceae)	*Radix*
JP Glycyrrhiza	*Glycyrrhiza uralensis* Fischer, or *Glycyrrhiza glabra* Linné *(Leguminosae)*	*Radix*
JP Gypsum	*Gypsum fibrosum*	-
JP Immature Orange	Citrus aurantium Linné var. daidai Makino, Citrus aurantium Linné, or Citrus natsudaidai Hayata (Rutaceae)	*Fructus immaturus*
JP Japanese Angelica Root	*Angelica acutiloba* Kitagawa, or *Angelica acutiloba* Kitagawa var. sugiyamae Hikino *(Umbelliferae)*	*Radix*
JP Jujube	*Zizyphus jujuba* Miller var. *inermis* Rehder *(Rhamnaceae)*	*Fructus*
JP Magnolia Bark	*Magnolia obovata* Thunberg (*Magnolia hypoleuca* Siebold et Zuccarini), *Magnolia officinalis* Rehder et Wilson, or *Magnolia officinalis* Rehder et Wilson var. *biloba* Rehder et Wilson *(Magnoliaceae)*	*Cortex*
JP Magnolia Flower	*Magnolia salicifolia* Maximowicz, *Magnolia kobus* De Candolle, *Magnolia biondii* Pampanini, *Magnolia sprengeri* Pampanini, or *Magnolia heptapeta* Dandy (*Magnolia denudate* Desrousseaux) *(Magnoliaceae)*	*Flos*
JP Mentha Herb	*Mentha arvensis* Linné var. *piperascens* Malinvaud *(Labiatae)*	*Herba*
JP Moutan Bark	*Paeonia suffruticosa* Andrews (*Paeonia moutan* Sims) *(Paeoniaceae)*	*Cortex*
JP Mulberry Bark	*Morus alba* Linné *(Moraceae)*	*Cortex*
JP Ophiopogon Tuber	*Ophiopogon japonicus* Ker-Gawler *(Liliaceae)*	*Radix*
JP Peach Kernel	*Prunus persica* Batsch, or *Prunus persica* Batsch var. *davidiana* Maximowicz *(Rosaceae)*	*Semen*
JP Peony Root	*Paeonia lactiflora* Pallas *(Paeoniaceae)*	*Radix*
JP Perilla Herb	*Perilla frutescens* Britton var. *crispa* W. Deane *(Labiatae)*	*Herba*
JP Peucedanum Root	*Peucedanum praeruptorum* Dunn, or *Angelica decursiva* Franchet et Savatier (*Peucedanum decursivum* Maximowicz) *(Umbelliferae)*	*Radix*
JP Pinellia Tuber	*Pinellia ternata* Breitenbach *(Araceae)*	*Tuber*
JP Platycodon Root	*Platycodon grandiflorum* A. De Candolle *(Campanulaceae)*	*Radix*

This is a provisional file, not the final typeset article

JP Pogostemon Herb	*Pogostemon cablin* Bentham *(Labiatae)*	*Herba*
JP Poluporus Sclerotium	*Polyporus umbellatus* Fries *(Polyporaceae)*	-
JP Poria Sclerotium	*Wolfiporia cocos* Ryvarden et Gilbertson (*Poria cocos* Wolf) *(Polyporaceae)*	-
JP Powdered Processed Aconite Root	*Aconitum carmichaeli* Debeaux, or *Aconitum japonicum* Thunberg *(Ranunculaceae)*	*Radix (processa)*
JP Pueraria Root	*Pueraria lobata* Ohwi *(Leguminosae)*	*Radix*
JP Scutellaria Root	*Scutellaria baicalensis* Georgi *(Labiatae)*	*Radix*
Lonicera Flower (Jinyinhua)	*Lonicera japonica* Thunb. *(Caprifoliaceae)*	*Flos*
Male Fern Rhizome (Mianmaguanzhong)	*Dryopteridis Crassirhizomatis* Nakai *(Polypodiaceae)*	*Rhizoma*
Mentha Herb (Bohe)	*Mentha* haplocalyx Briq. (Mentha canadensis Linné) (Labiatae)	*Herba*
Pinellina Rhizome processed with ginger (Jiangbanxia)	*Pinellia ternata* Breitenbach *(Araceae)*	*Tuber*
Pogostemon Herb (Guanghuoxiang/Huoxiang)	*Pogostemon cablin* Bentham *(Labiatae)*	*Herba*
Polyporus Sclerotium (Zhuling)	*Polyporus umbellatus* Fries *(Polyporaceae)*	-
Poria Sclerotium (Fuling)	*Wolfiporia cocos* Ryvarden et Gilbertson (*Poria cocos* Wolf) *(Polyporaceae)*	-
Roseroot (Hongjingtian)	*Rhodiola rosea* Linné *(Crassulaceae)*	*Radix et Rhizoma*
Scutellaria Root (Huangqin)	*Scutellaria baicalensis* Georgi *(Labiatae)*	*Radix*
Trichosanthes Seed	*Trichosanthes kirilowii* Maximowicz, *Trichosanthes kirilowii* Maximowicz var. *japonica* Kitamura, or *Trichosanthes bracteata* Voigt *(Cucurbitaceae)*	*Semen*
